# Computer Generated Realistic Interstellar Icy Grain Models: Physicochemical Properties and Interaction with NH_3_

**DOI:** 10.1021/acsearthspacechem.2c00004

**Published:** 2022-04-19

**Authors:** Aurèle Germain, Lorenzo Tinacci, Stefano Pantaleone, Cecilia Ceccarelli, Piero Ugliengo

**Affiliations:** † Dipartimento di Chimica, Università degli Studi di Torino, via P. Giuria 7, 10125, Torino, Italy; ‡ Institut de Planétologie et d’Astrophysique de Grenoble (IPAG), CNRS, Université Grenoble Alpes, rue de la Piscine 414, 38000 Grenoble, France; ¶ Dipartimento di Chimica, Biologia e Biotecnologie, Università degli Studi di Perugia, Via Elce di Sotto, 8, 06123, Perugia, Italy; § Nanostructured Interfaces and Surfaces (NIS) Centre, Università degli Studi di Torino, via P. Giuria 7, 10125, Torino, Italy

**Keywords:** Amorphous ice, GFN-xTB, GFN2, water clusters, adsorption, binding energy

## Abstract

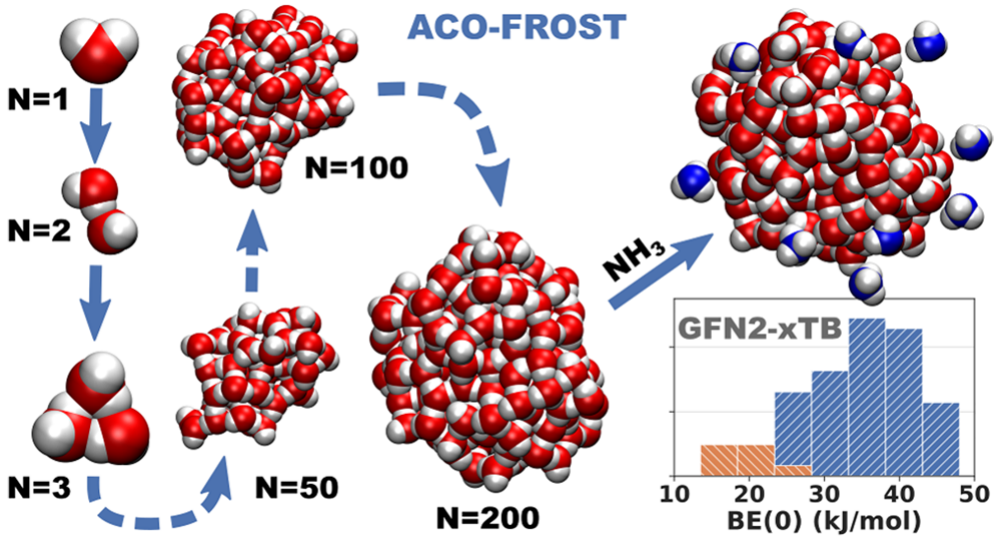

Interstellar grains are composed by a rocky core (usually amorphous silicates) covered by an icy mantle, the most abundant molecule being H_2_O followed by CO, CO_2_, NH_3_, and also radicals in minor quantities. In dense molecular clouds, gas-phase chemical species freeze onto the grain surface, making it an important reservoir of molecular diversity/complexity whose evolution leads to interstellar complex organic molecules (iCOMs). Many different models of water clusters have appeared in the literature, but without a systematic study on the properties of the grain (such as the H-bonds features, the oxygen radial distribution function, the dangling species present on the mantle surface, the surface electrostatic potential, etc.). In this work, we present a computer procedure (ACO-FROST) grounded on the newly developed semiempirical GFN2 tight-binding quantum mechanical method and the GFN-FF force field method to build-up structures of amorphous ice of large size. These methods show a very favorable accuracy/cost ratio as they are ideally designed to take noncovalent interactions into account. ACO-FROST program can be tuned to build grains of different composition mimicking dirty icy grains. These icy grain models allow studying the adsorption features (structure, binding energy, vibrational frequencies, etc.) of relevant species on a large variety of adsorption sites so to obtain a statistically meaningful distribution of the physicochemical properties of interest to be transferred in numerical models. As a test case, we computed the binding energy of ammonia adsorbed at the different sites of the icy grain surface, showing a broad distribution not easily accounted for by other more size limited icy grain models. Our method is also the base for further refinements, adopting the present grain in a more rigorous QM:MM treatment, capable of giving binding energies within the chemical accuracy.

## Introduction

Micrometer grain particles made of silicate and carbonaceous materials (core)^1,2^ are present in the interstellar medium (ISM). In dense molecular clouds, the low temperature (<20 K) and relatively high molecular density (≥10^4^ cm^–3^) favor the formation and subsequent freezing of molecules on top of these dust grains, forming amorphous layers of ice (aka mantles).^3−5^ Classical laboratory experiments using N_2_ adsorption isotherms on iced water revealed that, as a function of the formation conditions, micro porosity of the 2–3 nm size can be detected.^6^ The mantles are made mostly of water ice, formed by hydrogenation of oxygen atoms^7−9^ or by reactions of (OH)_2_ as studied by Redondo et al.,^10,11^ but molecules present in the gas phase (such as carbon monoxide) can also freeze onto the mantles of water ice.^3^ This richness of molecules make icy grains a host for important chemical reactions, such as the synthesis of H_2_^12^ (the most abundant molecule in the ISM^12,13^), dictated by the configuration of adsorption sites on the grain surface. The paths of formation of the most astrochemically relevant iCOMs have been recently reviewed.^14^ Other factors such as cosmic rays have been observed to compact the ice layers of interstellar grains.^15^ Hence, the study of adsorption, desorption, diffusion, and reaction of molecules on top of the icy grain surface is of high importance if we want to understand the evolution overtime of the chemical composition of the ISM. To study the atomistic details of these processes by computational chemistry methods we need icy grain models that embody the observed properties of interstellar icy grains.

As far as we know, no public grain models are available to the astrochemical community. Some models have been proposed in the literature, as summarized in the next few paragraphs, with the scope to satisfy requirements of specific cases, i.e., to study a given adsorbate or particular reaction features of interest. None of them was built with the purpose to have, in a single model grain, all the features exhibited by a real interstellar amorphous ice grain, i.e., in which adsorption sites exhibit different electrostatics, hydrogen bond features, and local dispersive interactions with respect to any adsorbed molecule.

The simplest model was proposed by Wakelam et al.,^16^ adopting a single water molecule as a model grain to compute the binding energies of dozens of chemical species at density functional level of theory (DFT), more precisely the M06-2X functional associated with the Gaussian aug-cc-pVTZ basis set. The computed BE are then corrected for the missing effects due to the adoption of a single water molecule, by linear fitting experimental BE against computed ones. The resulting global scaling factor brings the computed BEs closer to the experimental ones. Even though the procedure is clever and computationally very cheap, this approach cannot portray the richness of different binding sites at the grain and cannot provide the expected BE distribution.

Like Wakelam et al., Das et al.^17^ computed the BEs of a similar set of molecules using clusters from 1 up to 6 water molecules with the MP2 method and an aug-cc-pVDZ basis set. They showed a decreasing deviation between experimental and theoretical BEs by increasing the size of the water cluster. As for Wakelam et al. these clusters had, however, a limited number of hydrogen bonds per water molecules, decreasing the internal hydrogen bond cooperativity and the variety of binding sites.

To the opposite situation, grain models of hundred of thousands of water molecules have been built using special Lennard-Jones (LJ) potentials by Garrod^18^ and Christianson and Garrod.^19^ While these models match the interstellar icy grain sizes, they are not able to represent the hydrogen bond features linking each water molecules, due to the roughness of the adopted LJ potential. Furthermore, the size is so large that it is impossible to adopt DFT to characterize their electronic features.

In our group, we have also proposed both crystalline and amorphous ice models, within the periodic boundary conditions. In Zamirri et al.,^20^ three different periodic crystalline models were used to study the adsorption of CO on water ice using various DFT methods. However, the amorphous nature of the icy grains was missing. In Ferrero et al.,^21^ the adsorption of different molecules was studied on periodic crystalline and amorphous water ice models using DFT (B3LYP-D3/A- VTZ* and M06-2X/A-VTZ*). The amorphous unit cell included 60 water molecules allowing to identify from three to eight different binding sites as a function of the considered adsorbate molecule, providing some variability in the computed BE values. In that work, however, the adsorbates were manually positioned at the ice sites to maximize the hydrogen bond strength, likely biasing the final values of the BEs.

Sameera et al.^22^ used a crystalline ice structure to represent icy grains surfaces and computed the BE of several radical species such as CH_3_. Up to 16 crystalline models with approximately 160 water molecules exhibiting 16 different binding sites, were built up using the crystalline water ice structure. The clusters were treated with a QM:MM method containing at least 44 H_2_O for the QM (wB97XD/def2-TZVP) part and 112 for the MM (AMBER force field or the AMOEB09 polarizable force field) part. In the same work, amorphous solid water cluster models of 162 H_2_O molecules exhibiting up to 10 different binding sites were also used to study CH_3_ radicals with a QM:MM method (49 molecules for the QM part and 113 for the MM part).

Shimonishi et al.^23^ used nine amorphous water clusters including 20 water molecules (optimized with ω-B97XD and the Gaussian 6-311+G basis set) to represent different binding sites that could be found on the grain, even if the sizes are still rather limited to provide enough variability. Rimola et al.,^24^ Duvernay et al.,^25^ and Enrique-Romero et al.^26^ all used water clusters ranging from 18 to 32 water molecules and various DFT methods to study chemical reactions bringing simple radical species to iCOMs. In that case, due to the highly demanding cost of characterizing the entire potential energy surface of the reactions, the site variability was less relevant than for computing the BEs. Bovolenta et al.^27^ used two types of clusters made of 22 and 37 water molecules (revPBE0/def2-TZVP) to study the adsorption of HF and found minimal changes in the BE distributions between the two cluster sizes.

Last, Song and Kästner^28^ proposed an icy model (also used in Lamberts and Kästner,^29^ Molpeceres et al.,^30^ and Molpeceres and Kästner^31^) as an hemisphere of 499 water molecules in which 295 are free to react, cut out from a 18937 water molecules cell that was equilibrated at 300 K for 100 ps and then suddenly quenched at 10 K for 50 ps using the NAMD code (in the *NVT* ensemble and using a Langevin thermostat). This model is close to the one we are proposing here, but its construction does not have any resemblance with the process occurring in the ISM in which water is added step by step at a very low temperature.

The interested reader can found more about modeling interstellar ice in the recent papers by Zamirri et al.^14^ and Cuppen et al.^32^

In this paper, we will describe a new approach to automatically building up an amorphous icy grain model constituted by several hundreds of water molecules. We labeled our models as “realistic”, not based on a size criterion only but requiring the following essential features: (i) the grain should not be minimal, i.e., envisaging 20–30 water molecules only, as usually found in the literature, but includes at least few hundreds water molecules; (ii) the hydrogen bond features within the icy grain should be accurately represented by adopting a proper quantum level of theory; (iii) the ice should be amorphous by construction, avoiding the usual approach of heating at high *T* and sudden cooling of a crystalline ice model, which has no counterpart in the grain evolution in the ISM. Indeed, the grain is not derived from crystalline ice or the like, but is built from scratch by sequentially adding water molecules in a way comparable to ice generation in terrestrial laboratories. In the ISM conditions, as already discussed, water is formed *in situ* and we will provide the reasons why this process is very hard to be simulated quantum mechanically (*vide infra*). As for the adopted computational method, we choose the newly developed xTB semiempirical quantum mechanical GFN2 method^33^ and the force field GFN-FF,^34^ both developed by Grimme’s group at the University of Bonn. Both methods showed excellent results with system dominated by noncovalent interactions.^34−38^ In previous papers, we also showed that GFN2 was close in accuracy to CCSD(T)/CBS for small water clusters^39^ and very accurate when compared to DFT methods for the computation of binding energies of several molecules adsorbed on a crystalline water ice slab,^40^ making them well suited for our purpose. On the generated icy grain, we showed how to compute the BEs of ammonia (NH_3_) on more than 100 different adsorbing sites of a 200 water icy grain, providing the BE distribution at very low computational cost with reliable BE values.

## Computational Details

### Methodology

All the calculations were carried out with the xTB program^41^ (version 6.3.3 for the binding energy sampling of NH_3_, and 6.4.0 for the icy grain model of 1000 water molecules), using two different methods: the semiempirical GFN2^33^ and the classical force field GFN-FF.^34^ For the grain building process, all geometry optimizations at GFN2 and GFN-FF levels were performed using the default settings of the code (i.e., 5 × 10^–6^ hartree and 1 × 10^–3^ hartree bohr^–1^ on energy and gradient, respectively) while molecular dynamics simulations were done only with GFN-FF with the temperature set to 10 K to mimic the ISM conditions, a time step of 1.0 fs, and a total time of 1 ps. For the BE distribution, all calculations were performed using GFN2 and the default settings except for the precision of geometry optimizations that was set to “extreme” (i.e., 5 × 10^–8^ hartree and 5 × 10^–5^ hartree bohr^–1^ on energy and gradient, respectively). Constraints on fixed atoms were used through the keyword ”constrain” which apply a force constant of 0.5 hartree bohr^–1^ to the distance among selected atoms.

The different action performed for the grain building process and the binding energy sampling explained in the following sections were automated via two Python scripts combining approximately two thousands lines of codes and using standard packages such as Numpy and Scipy, and more specialized ones such as the Atomic Simulation Environment (ASE). The set of scripts named ”ACO-FROST” are freely available (see Data and Software Availability).

The BEs of NH_3_ at the grain were computed as follows:
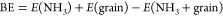
1Here *E*(NH_3_), *E*(grain), and *E*(NH_3_+grain) are the GFN2 electronic energies after geometry optimization of NH_3_, the water grain with the constraints applied (*vide infra*), and NH_3_ interacting with the grain, respectively.

To confirm that the optimized geometries are minima on the potential energy surface, we computed the harmonic vibrational frequencies and checked that all frequencies were real-valued. Structures with imaginary frequencies were discarded. Careful check of each of these structures revealed that the imaginary frequency belongs to atoms of the local region where NH_3_ is adsorbed.

From the set of frequencies, the zero point energy (ZPE) is computed for each component to arrive to the zero point energy correction (ΔZPE):
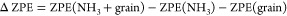
2to compute the zero point energy corrected binding energies BE(0), by subtracting the ΔZPE to the electronic BE:
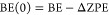
3

In general, the ΔZPE is a positive quantity; therefore, the BE(0) is always smaller than the electronic BE.

To characterize the shape of the generated icy grains we computed the gyration tensor as described in Theodorou and Suter.^42^ For a three-dimensional Cartesian coordinate system, it reads:
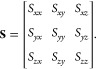
4

With each component *S*_*mn*_ of the *S* matrix as
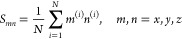
5with *N*, the number of atoms in the system, and *m*^(*i*),^ the Cartesian coordinate *m* = *x*, *y*, *z* of the *i*th atom.

By diagonalizing the **S** matrix the eingenvalues λ_*x*_^2^, λ_*y*_^2^, and λ_*z*_^2^ are ordered as λ_*x*_^2^ ≤ λ_*y*_^2^ ≤ λ_*z*_^2^. From them, we derived the gyration radius *R*, the asphericity *b*, and the relative shape anisotropy κ^2^ defined as
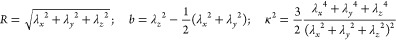
6

The asphericity *b* is greater than zero, becoming exactly zero only when λ_*x*_ = λ_*y*_ = λ_*z*_ (spherically symmetric system). The relative shape anisotropy κ^2^ is bounded between zero (all points are spherically symmetric) and one (all points lie on a line).

Hydrogen bonds were sampled by computing the O···H distances of the GFN-xTB optimized structures and imposing a lower/upper threshold of 1.2/2.2 Å to filter out the O···H bonds. Dangling hydrogens were sampled by counting every hydrogen without an O distance inferior to 2.2 Å; dangling oxygens are defined as oxygen atoms free from an O···H distance smaller than 2.2 Å. To classify dangling hydrogen/oxygen atoms a minimum distance from the structure center of mass was forced to limit the influence of possible dangling species present inside the structure.

We also computed the density ρ of each grain by assigning van der Waals radii to H/O of 1.30/2.05 Å and using a Monte Carlo integration scheme (using 30000 sampling points) to compute the corresponding grain volume as encoded in the MOLDRAW program.^43^ The adopted radii were chosen so that the computed density ρ of the experimental structure of crystalline ice *I*_*h*_ resulted in the experimental^44^ value ρ = 0.92 gr/cm^3^.

We also render many of the static structures shown in the figures as hyperactive molecules using the JSmol rendering engine.^45^ These were made accessible through specific QR codes readable through the standard mobile phone camera.

### Grain Building Process

Water can be formed by gas-phase reactions. However, by far the largest fraction of the total amount of water in cold (≤200 K) ISM comes from reactions between oxygen and hydrogen atoms at both the grain core and on top of an already formed icy mantle.^46^ Simulating this process is, however, computationally very expensive and almost impossible to carry out on icy clusters of large size. To overcome this difficulty, we assume that the water molecules are already formed and that they are adsorbed on top of an initial seed (a single water molecule). It is worth noting that laboratory experiments meant to simulate the ISM ice grow the grain by addition of preformed water molecules adsorbed on specific inert supports. This implies that the energy transfer between the newly formed OH bonds toward the grain, with possible restructuring of the ice, is missing within this approach. However, recent studies from our lab (Pantaleone et al.^47,48^) at *ab initio* molecular dynamics level of the H_2_ and HCO radical formation on both crystalline and amorphous ice models (extended models within the periodic boundary conditions) did not show signs of amorphization of the ice, irrespective of its initial degree of crystallinity. Nevertheless, we decided to amend for the potential missing energy transfer by running a short MD computation at 10 K at given steps during the ice accretion (*vide infra*).

**Figure 1:**
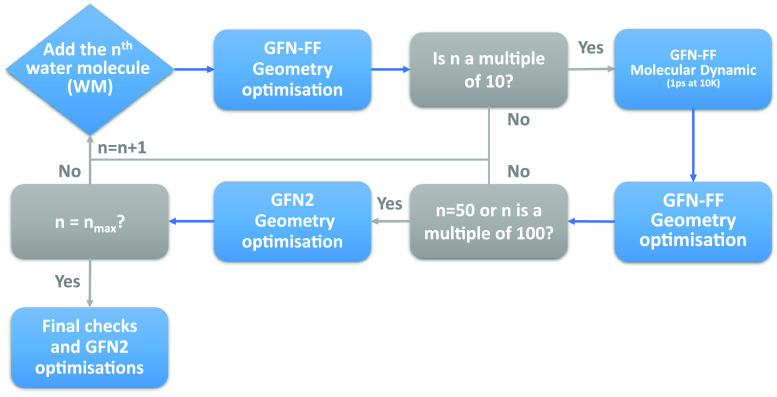
Flowchart of the grain building process (a video showing the building process of the 1000 water molecules grain is available in the Supporting Information).

The grain building process is iterated in a series of sequential steps adding water from a random direction, internal orientation and distance from the grain surface (between 2.5 and 3 Å, slightly more than the typical H-bond length), until the desired number of water molecules is reached. A schematic representation of the procedure is summarized in Figure 1. At each new added water molecule the structure is first optimized at GFN-FF level. As discussed before, to simulate the conditions of the ISM in which a fraction of the heat of the water formation is transferred to the grain increasing its local temperature,^47^ every 10 added water molecules a *NVT* molecular dynamics at 10 K is performed for 1 ps to simulate the water relaxation, directly followed by a GFN-FF optimization. We counted, on average, from a sample of 20 clusters of 200 water molecules, an increase of 6 hydrogen bonds as a result of the MD step followed by a GFN-FF optimization. One drawback of GFN-FF is its tendency to produce ice structures which are too fluffy, with many water molecules with incomplete H-bond coordination behaving like dangling species. To avoid this nonphysical feature, at regular intervals during the grain building process, a GFN2 full optimization is performed. At variance with GFN-FF, GFN2 shrinks the size of the cluster, to maximize the hydrogen bond (H-bond) interactions. This allows water molecules to form a compact structure which is preserved even after the subsequent GFN-FF optimizations (see Figure S1 of the Supporting Information). This strategy ensures the best compromise between speed and quality of the intermolecular interactions in the formed grain. Table 1 shows the comparison of the computational time between the two methods for a 200 water cluster.

**Table 1:** Comparison between the GFN2 and GFN-FF Geometry Optimization Times for Different Grain Sizes^a^

Number of water molecules	GFN2	GFN-FF	ratio GFN2/GFN-FF
200	34 min	1 min 26 s	24
400	5 h 15 min	7 min	44
800	1 day 17 h	53 min	46

a The xTB program was run on 20 core Intel Xeon E5-2630 v4 @ 2.20 GHz.

When the desired number of water molecules is reached a final GFN2 full relaxation is performed, followed by an automatic quality check on the grain structure aimed at correcting any geometrical oddity that could have arisen during the grain building, such as water molecules interacting with a single H-bond. If found, these water molecules are removed, and the process goes back a few steps by removing the upper water layers and to reconstruct again the grain until no geometrical oddities are present and the number of desired water molecules is reached.

### Binding Energy Sampling

To compute the binding energy distribution of NH_3_ a grain consisting of 200 water molecules was used, as it represents the best trade-off between the number of binding sites (large enough to have a significant statistical distribution) and the computational cost.

To sample all possible adsorption sites of NH_3_ around the grain model, a discrete grid of points taken from Teanby^49^ was superimposed to the grain surface, where each point represents the NH_3_ barycenter. In this way, a uniform and complete coverage of NH_3_ molecules around the icy grain was achieved, ensuring an unbiased BE sampling of strong and weak binding energies.

**Figure 2:**
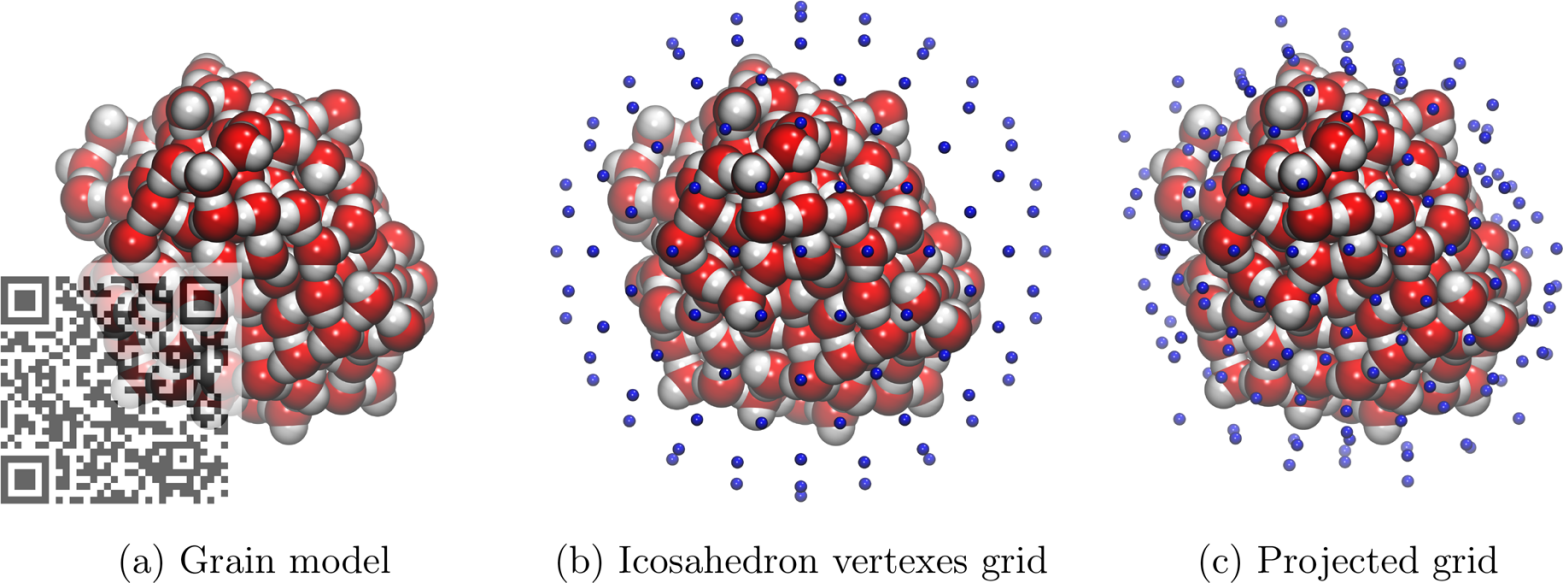
Van der Waals representation of the icy grain (section a); starting grid positions (as blue dots, section b) and final projected grid for the location of each NH_3_ molecule (JSmol views can be seen by scanning the overlapping QR code).

The grid is initially made up of 12 vertices of an icosahedron and serves as starting position for NH_3_. These 12 initial vertices can then be duplicated several times to obtain additional subgrids with an increased number of vertices. This implies that the vertices of a subgrid will be also included in all subsequent tighter levels, without the need to recalculate the points of the previous level (see Figure S3 of the Supporting Information). Figure 2 shows the bare grain, and the grid level used for the NH_3_ BE sampling. It contains 162 vertices which are used as barycenter for NH_3_ and then projected on top of the grain structure. The projection brings a distance between 2.5 and 3 Å from the grain, used for NH_3_ positioning. For each positioning, NH_3_ is also randomly rotated to ensure an unbiased contact with the grain surface. The BE is computed for each position (blue dot) occupied by NH_3_, resulting in 162 different values (*vide infra*).

**Figure 3:**
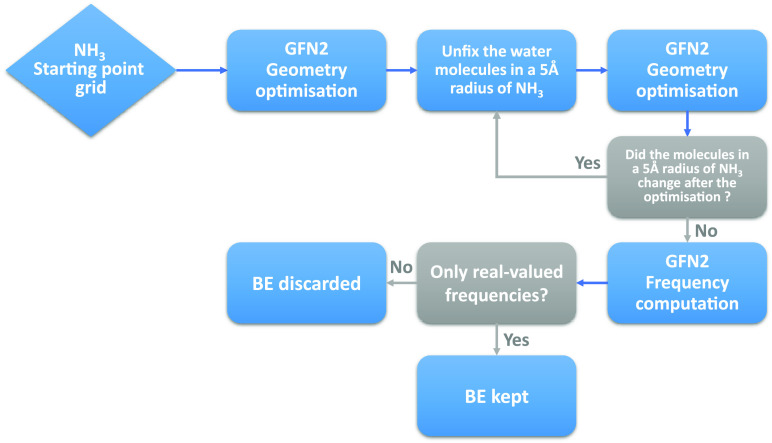
Flowchart of the NH_3_ BE distribution sampling.

Once the starting positions of NH_3_ have been defined, the method to compute the BE consists of two geometry optimization steps using GFN2 (see Figure 3). At the first step, only the coordinates of NH_3_ are allowed to relax, while the coordinates of all the water molecules composing the grain are held fixed at their starting positions. This strategy ensures that, despite the relatively small grain size, the long-range structural rigidity of the real grain is enforced by limiting the mobility of the model. This strategy ensures also a unique reference for the free ice cluster, in agreement with what would happen for a very large and more realistic cluster. In a second step, in line with the previous strategy and considering the interaction of NH_3_ as a local one, we relaxed, together with that of NH_3_, the coordinates of the water molecules within a 5-Å radius from the NH_3_ location. This last relaxation process can, in principle, displace the NH_3_ molecule from the starting position and, therefore, reaching a position in which new interactions with water molecules not included in the original relaxation sphere have to be considered. To account for these cases, we redefined a new sphere of 5 Å around NH_3_: if the number of water molecules included in the new sphere changes with respect to that included in the original sphere a new GFN2 optimization is carried out. This process is repeated until the number of water molecules included in the sphere does no longer change during the optimization procedure. One possible drawback of this procedure is that the NH_3_ molecule starts to travel on the grain surface at each new defined sphere, moving far apart from the original position. To mitigate this problem, we discarded situations in which the sphere of neighbors has to be redefined more than twice. Fortunately, we observed only 21 of such cases. Harmonic frequency calculations were run on the optimized structures to ensure that the final structures were indeed a minimum on the constrained potential energy surface (PES). All the structures giving one or more imaginary frequencies were rejected from the BE distribution.

## Results

### Icy Grain Model

**Figure 4:**
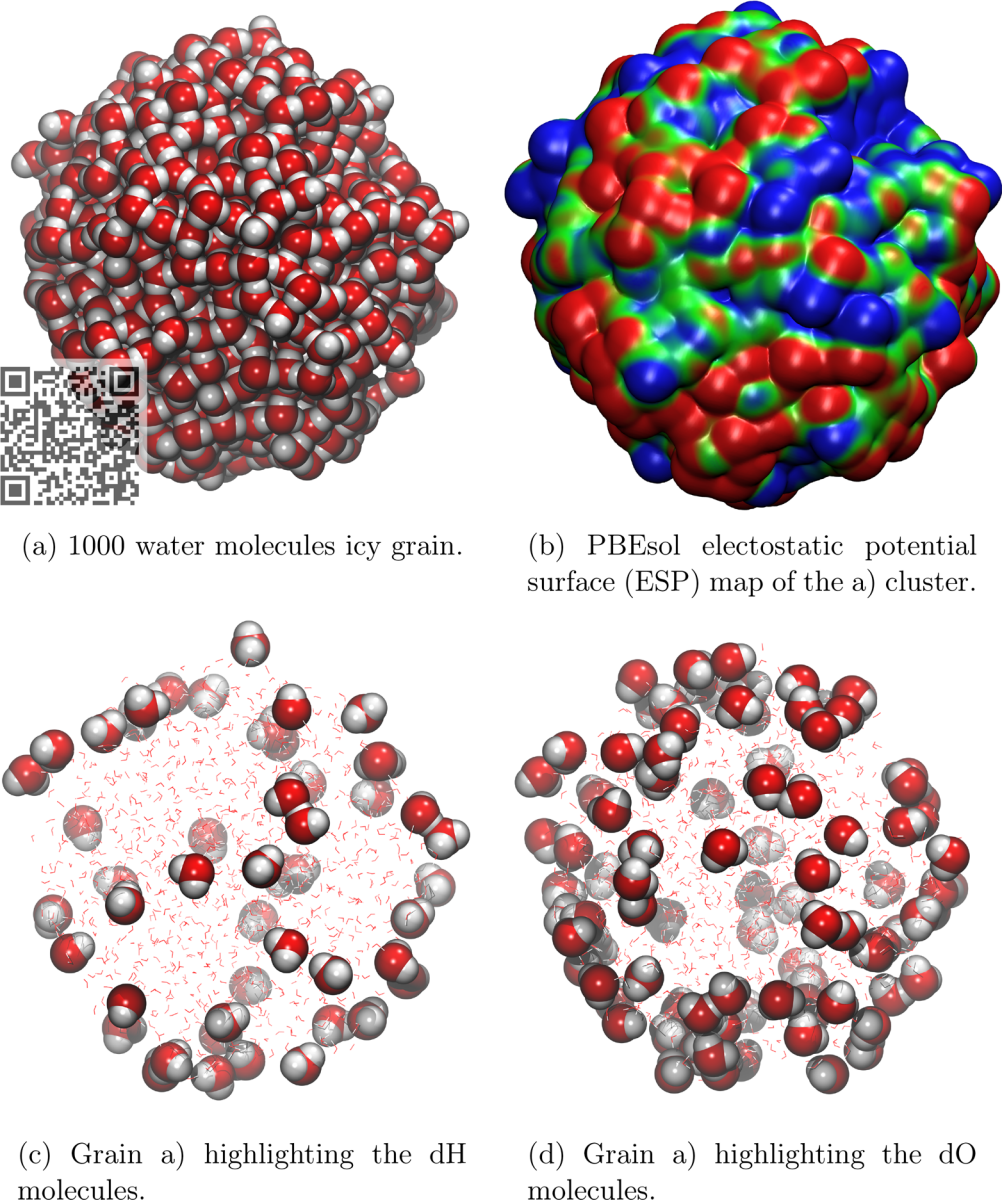
Different representation of a 1000 H_2_O molecules grain highlighting: (a) the surface sites structure (a JSmol view can be seen by scanning the overlapping QR code); (b) the electrostatic potential (blue (positive)/red (negative) zones); (c and d) the dH (dangling hydrogen) and dO (dangling oxygen) sites.

**Figure 5:**
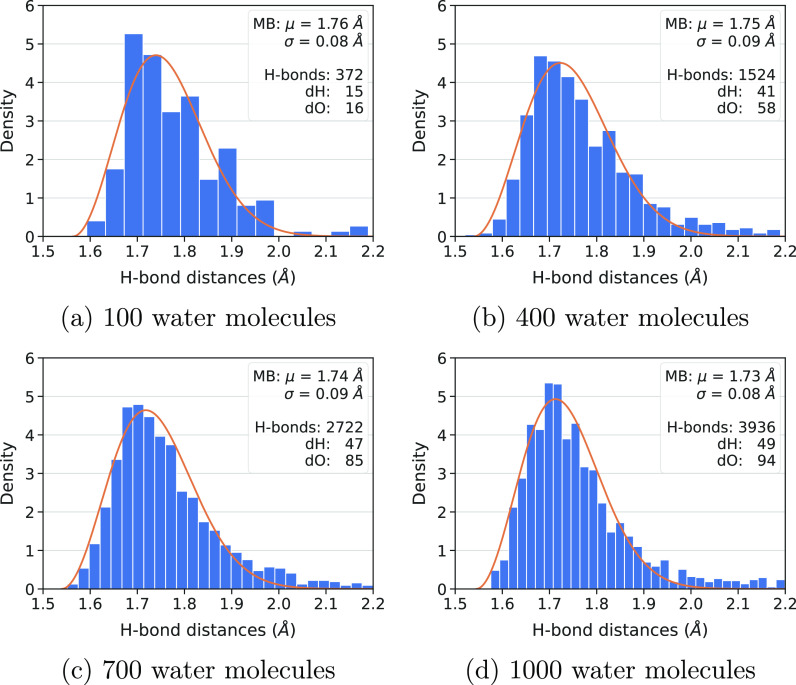
H-bond length (O···H) distribution of four icy grains of different size. Top right inset to each plot: average H-bond length μ and its standard deviation σ of the Maxwell–Boltzmann distribution fit; sum of the number of H-bonds given and received for each H_2_O molecule; number of dH (dangling hydrogen) and dO (dangling oxygen) sites.

**Figure 6:**
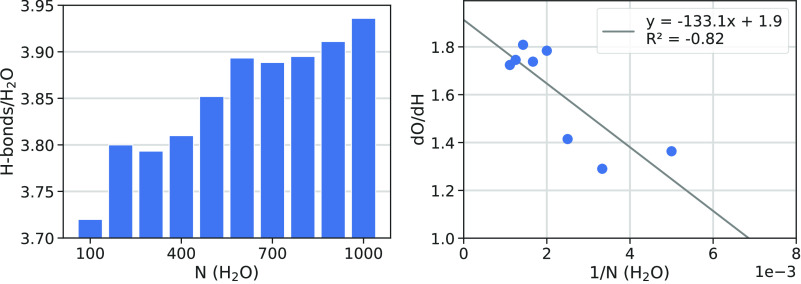
Number of H-bonds received and given per water molecule for different grain sizes (left). Ratio of dangling oxygens (dO) and hydrogens (dH) for the different grain sizes (right).

The above-described procedure was carried out to build up a cluster up to 1000 water molecules. We extracted selected structures characterizing, as a function of the cluster size, the following features: H-bond length distribution (Figure 5), number of H-bonds per water molecule (Figure 6, left) and ratio of the number of dangling oxygens (dO) over the number of dangling hydrogens (dH) as a function of the inverse of the grain sizes in water molecules (Figure 6, right). Figure 4 shows the structure (in the van der Waals representation), the electrostatic potential (ESP) map of the 1000 water grain, and the vdW representation of the dangling species dH and dO (Figure S4 of the Supporting Information highlights multiple amorphous surface structures of the grain). The ESP has been computed as a single point calculation at PBEsol^50^ using the parallel version of CRYSTAL17.^51^

The H-bond length distribution for different grain sizes is nicely fitted by a Maxwell–Boltzmann probability density function (Figure 5). The feature of the distributions are all very similar as a function of the grain size, with the average H-bond length in the 1.73–1.77 Å interval, showing rapid convergence of the H-bond features with the cluster size.

The average of the total number of H-bonds per H_2_O molecule starts at 3.72 (100 water molecules) up to 3.94 (1000 water molecules) (Figure 6, left), indicating that almost all water molecules of the grain behave as a regular water in bulk ice (four H-bonds per water molecule). Obviously, water molecules at the grain surface contribute to lower the full H-bond coordination as both dangling hydrogen (dH) and oxygen (dO) are present at the grain surface. Both dH and dO sites are extremely important for the adsorption of H-bond acceptor (like NH_3_) and H-bond donor molecules (like CH_3_OH), respectively. The ESP map of Figure 4 highlights the differences in terms of the electrostatic potential associated with the two dangling species. The total number of dangling species increases with the cluster size (see Figure 5), as well as the dO/dH ratio to reach 1.9 at infinite cluster size (see Figure 6, right). Interestingly, the number of dO is almost twice as large as that of dH, which is reflected by the ESP map, whose surface we estimated roughly to be occupied at 46% by negative (red-dO) zones, 38% by positive (blue-dH) zones, and the remaining 13% by almost neutral (green) zones. This distribution has important consequences on the capability of the ice grain to preferentially adsorb molecules with a H-bond donor character with respect to molecules with an H-bond acceptor character.

The density ρ of the *N* = 200, 500, and 900 grains was computed to be 1.12, 1.21, and 1.25 gr/cm^3^, respectively, with an error of ±0.03 gr/cm^3^. This shows an increased density of the grain with respect to that of the crystalline ice of ρ = 0.92 gr/cm^3^, in agreement with the amorphous (liquidlike) character of the simulated grains. However, in absence of a GFN2 calculation of a crystalline ice (at the moment periodic boundary conditions calculations are not yet implemented in the xTB code), some uncertainty in the ρ absolute values due to the systematic errors of GFN2 is expected.

**Figure 7:**
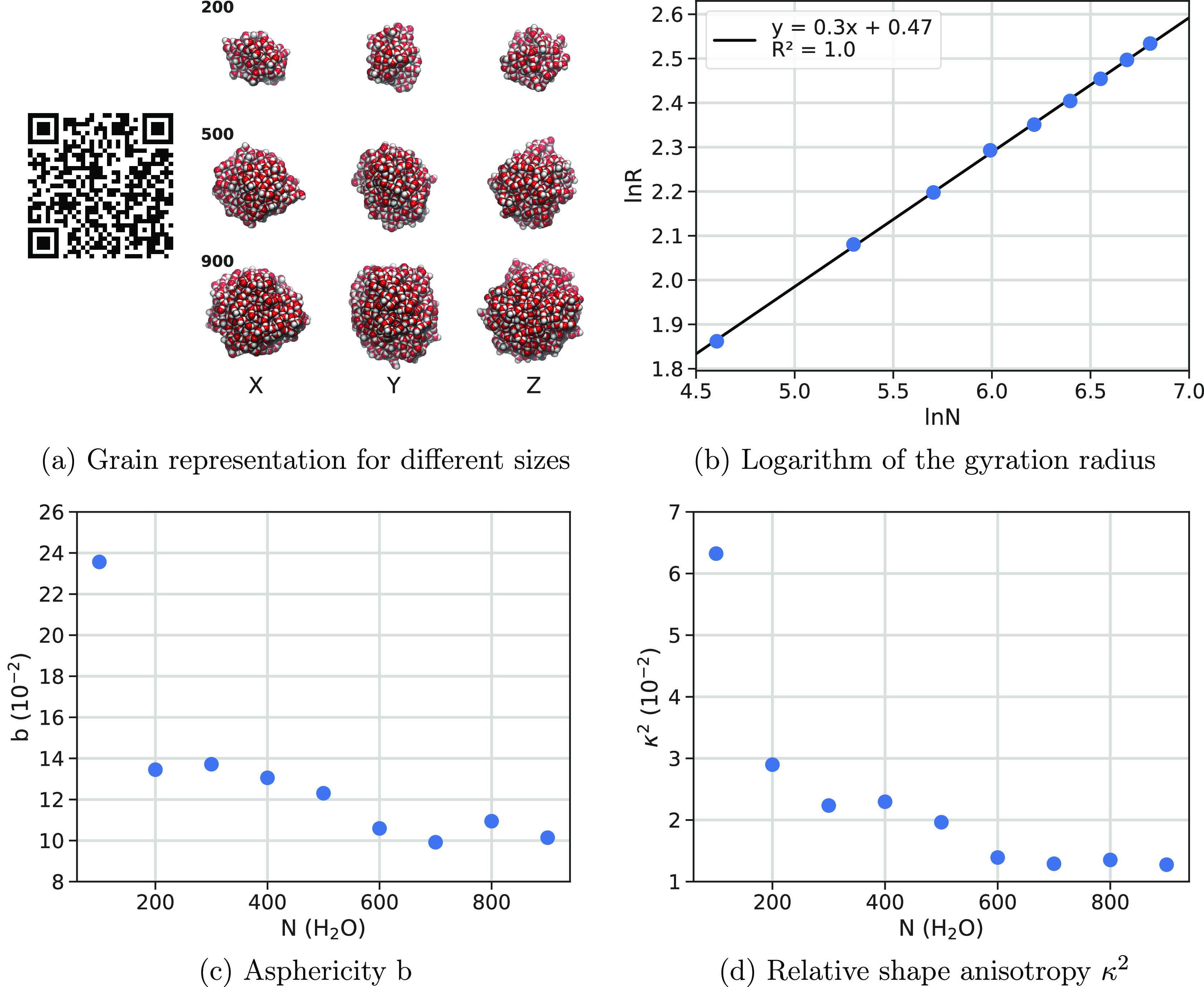
Different shape descriptors from the gyration tensor for different grain sizes (JSmol views can be seen by scanning the QR code). In part a, *X*, *Y*, and *Z* represent the view axis facing the screen.

The shape of the derived grains has been characterized by the gyration tensors and related quantities (see the Computational Details). Figure 7b shows a very nice linear correlation between the natural logarithms of the gyration radius R and that of the size *N* of the grain. The slope close to ^1^/_3_ can be justified by considering that the volume of the grain associated with the gyration radius is *V* = ^4^/_3_π*R*^3^ ≈ *v*_w_*N*, in which *v*_w_ is the gyration radius associated with a single water molecule. By taking the natural logarithm of both sides, one gets the dependence of ^1^/_3_ ln(*N*), close to the best fit value. The graph warns that, if one doubles the *R* value of a 1000 water cluster (from 13 to 26 Å), the number of needed water molecules increases by 1 order of magnitude, implying a steep increase of the computational resources needed to simulate grains of realistic size. Both the asphericity (Figure 7c) and the relative shape anisotropy (Figure 7d) data show a rapid convergence toward very small values, reminiscent of the increased spherical shape of the grain with the increased cluster size as visually shown in Figure 7a.

**Figure 8:**
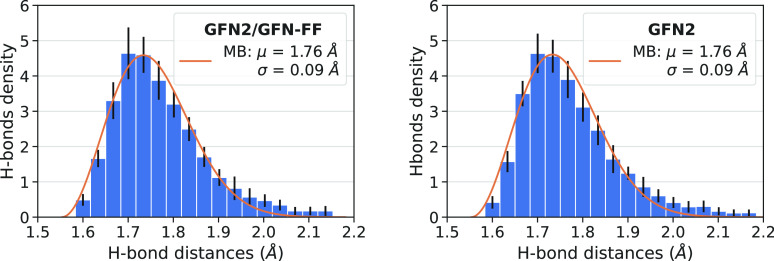
Average H-bond length for the 20^th^ water clusters made up of 200 water molecules. The building process was carried out at both GFN2/GFN-FF (left) and at full GFN2 (right) levels. Black lines represent the standard deviation, and the orange line represents the Maxwell–Boltzmann fit.

Due to the random positioning of each added water during the grain accretion, different starting random seeds used to initiate the pseudorandom numbers will lead to different grains morphology. To explore such a dependence, we run 20 models with the same final composition of 200 water molecules using different initial random seeds. Averaging the resulting structural features for all considered grains gives 379 ± 4.4 number of H-bonds, 21.4 ± 3.1 dH, and 30.6 ± 2.8 dO. We also computed the H-bond distances (H···O) and averaged them to obtain a distribution over the 20 grains, for each icy grain model (Figure 8, left). The small standard deviation of all these properties, as well as the similarity of the H-bond distribution, make us confident that the grain models should have the same characteristics. Therefore, we expect similar BE distribution for grains obtained with different initial random seeds. As Figure 8 (right panel) shows, there is almost no difference by building up the cluster with the faster strategy of adopting a GFN-FF first optimization followed by a GFN2 final optimization step with respect to a more expensive full GFN2 optimization, giving credit to the adopted building up strategy. As already pointed out, this strategy allows a huge saving of computer resources (see Table 1).

### Binding Energy Sampling

Binding energies of NH_3_ molecule on a 200 water molecules grain (a vdW representation of the grain and an EPS map are shown on Figure S5 of the Supporting Information) were computed using the method depicted in Figure 3. Ideally, the largest cluster including up to 1000 water molecules would be a better option than the one with 200 water molecules. For instance, Figure 6 shows that the dO/dH ratio for the 200 water molecules cluster is underestimated with respect to the largest one. Nevertheless, we will show later (see Figure 11) that clusters with the same size but different dO/dH ratio are giving NH_3_ BE distributions which are very close to each other. Considering that one BE evaluation on the largest cluster would take a full day long to accomplish and that more than 100 different cases should be run for the BE distribution, the adoption of the 200 water molecules was a reasonable trade-off between the computational cost and the number of available adsorption sites. This was also enough to ensure a representative statistical sampling of NH_3_ at the surface sites. Clearly, future work is needed to assess the variability of the BE distribution as a function of the ice cluster size.

**Figure 9:**
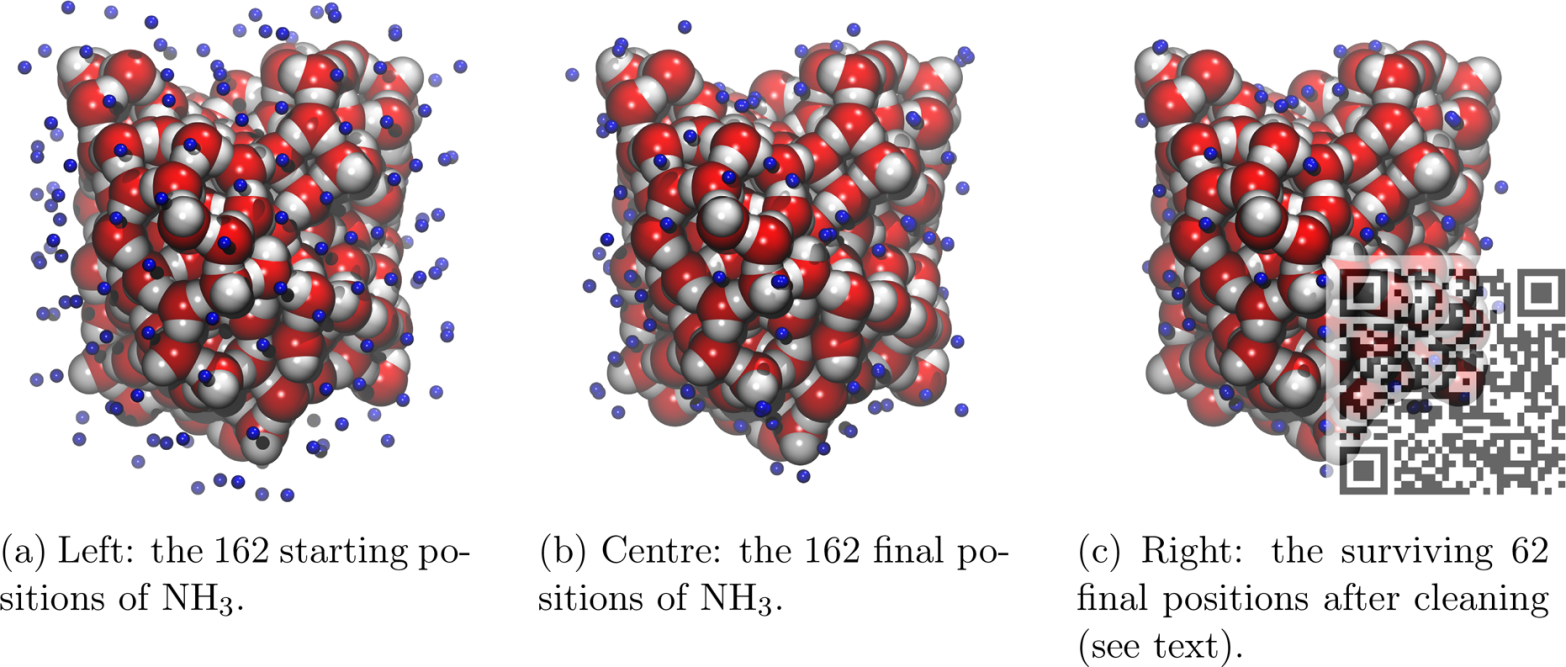
Positions of the NH_3_ molecules at different stages of the BE calculations (JSmol views can be seen by scanning the overlapping QR code of part c).

**Figure 10:**
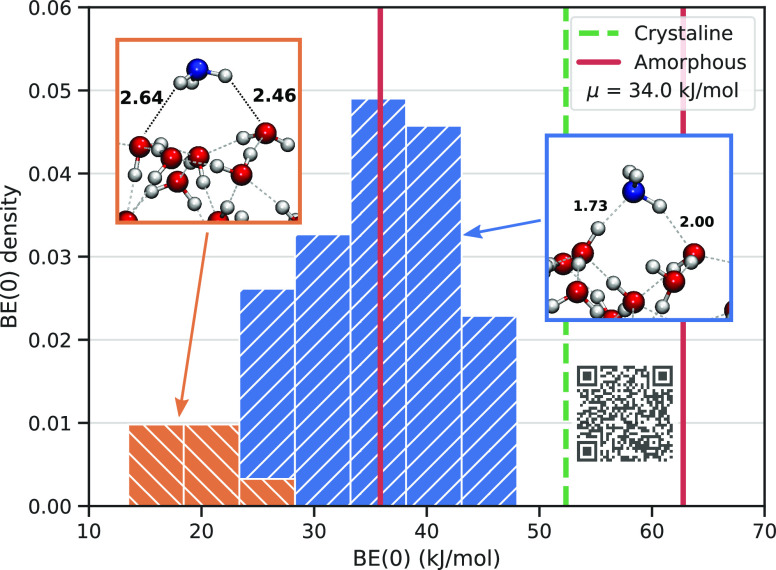
NH_3_ zero point energy corrected BE distribution (JSmol representative structures can be seen by scanning the overlapping QR code).

On each sample, several checks were performed to discard those showing the following features: (i) presence of imaginary frequencies; (ii) nonconservation of the number of water molecules within the 5 Å-radius for more than two runs during the optimization procedure (see the Computational Details); (iii) duplicated structures. Of the 115 samples left after criteria i and ii, 31 land to a minimum which is present only once in the BE distribution, while the remaining 84 samples collapse to the same minimum of at least one other structure. More specifically, 19 structures present two twin minima, 7 three, 3 four, 1 six, and 1 seven (see Figure 9). Therefore, a total number of 62 binding sites were finally selected in the computed BE distribution of Figure 10. In the Supporting Information we discussed shortly the changes between the fixed and partially unfixed final structures and their respective BE distribution. It may be possible that more binding sites could emerge by starting with a tighter grid for the NH_3_ positioning, but it is unlikely that the number increases significantly. As one can see from Figure 10, NH_3_ acts as both H-bond donor and acceptor; compared to H_2_O, NH_3_ is a much stronger H-bond acceptor but a weaker H-bond donor. The most stable interactions are those where NH_3_ is both a H-bond donor and acceptor, while the least stable ones exhibit NH_3_ as exclusively H-bond donor, as represented by the orange histograms of Figure 10. With the approach used throughout the paper, also weakly bounded systems are taken into account, because of the starting random orientation of NH_3_. These cases are particularly interesting for the potential chemical reactions occurring in the ISM, because the weaker the BE of the adsorbate is with the surface, the higher its probability to diffuse and therefore to react with other neighboring species adsorbed at the grain surfaces is.

In Figure 10 we also report the BE values of NH_3_ computed by Ferrero et al.^21^ on periodic models of either crystalline (dashed line) and amorphous (full lines) ices. Crystalline ice presents only one BE because the dO and dH distribution is controlled by the imposed crystal symmetry and, accordingly, only one adsorption site is found for the NH_3_ case. However, crystalline ice is not the dominant water ice phase, even though it was found in non-negligible abundance in protoplanetary disks^52^ and in protostellar molecular shocks.^53^ Therefore, crystalline ice does not represent a realistic model of a typical grain ice mantle, usually of amorphous nature. The periodic amorphous model developed by Ferrero et al.^21^ is large enough to identify different sites, but still too small to allow the building up of a sensible BE distribution. The BE range reported by Ferrero et al. falls at higher values with respect to ours. We suspect this is due to two reasons: (i) first, the rigidity of our cluster compared to the full relaxation allowed by the periodic amorphous slab; (ii) second, we do not bias the positions of the NH_3_ molecule, while Ferrero et al. set up its position manually by maximizing the chance of a strong H-bond interactions.

**Figure 11:**
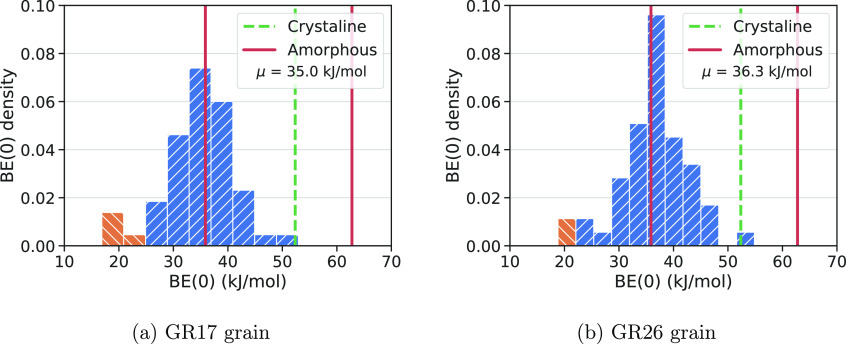
NH_3_ BE(0) distributions for GR17 and GR26 clusters grains.

To obtain a more reliable BE distribution, the adsorption of NH_3_ was studied also on two different grains among those generated with the different random seeds. The new grains have been chosen according to the largest difference in the number of dH, while the dO remains almost the same. The chosen GR17 and GR26 models identify grains with dO/dH(GR17) = 30/17 = 1.76 and dO/dH(GR26) = 32/26 = 1.23, respectively. Figure 11 shows the NH_3_ BE distribution for these two grains. Both grains present an identical number of adsorption sites, which, after proper cleaning (*vide supra*), is 54, allowing for a fair comparison. We expect that GR26, with a higher dH, will give higher BEs due to the nature of NH_3_ as H-acceptor molecule. However, this is not the case: the average BE on the two grains is very similar (35.0/36.3 kJ/mol for GR17/GR26), with corresponding BE minima/maxima at 16.9/18.8 kJ/mol and 52.9/54.9 kJ/mol. Despite the very modest increase in the BE for the GR26, all values are almost grain model independent, thus showing convergence of data on the final shape of the grain at a fixed size. A careful analysis of the BE distributions on Figure 11 reveals a difference in the shape at high BE values with an overrepresentation of values around 35 kJ/mol for GR26 compared to GR17, while the tail at low BE values is rather similar (orange part of the chart), in agreement with the excess of dH of GR26 over GR17.

**Figure 12:**
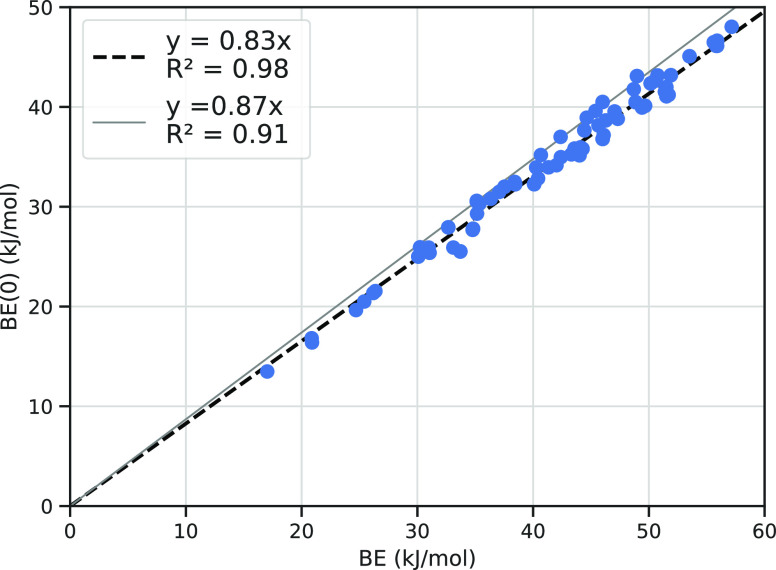
Linear correlation between BE and zero point energy corrected BE(0) values (kJ/mol) at GFN2 level from a previous work^40^ (continuous line) and for all considered cases of NH_3_ adsorption at the 200 water grain of the present work (best fitted dashed line on filled circle points).

One important issue is to correct the electronic BE for the zero point energy (ZPE) contribution (see Computational Details), the most important correction to the BE at the very low temperature of the interstellar medium. However, the calculation of the Hessian matrix (second derivative of the GFN2 energy with respect to each nuclear Cartesian coordinate) is very expensive and it would be useful to find a way to skip this step. For instance, while the Hessian GFN2 calculation for a 200 water grain takes half an hour, about 6 days are needed to compute the 800 water grain (time obtained on the same hardware reported in Table 1). Here, we propose a simplified method to avoid this expensive step, by using our recent work^40^ in which we compared the GFN2 BE with the *ab initio* ones by Ferrero et al.^21^ for a large set of molecules adsorbed on crystalline models of ice. There, we also compared the GFN2 BE against the ZPE BE(0) corrected one, for each considered molecules, including NH_3_. Figure 12 shows the best linear correlation (continuous line) using our previous data contrasted with the present GFN2 BE and BE(0) ones (dashes) for all considered NH_3_ adsorption cases at the icy grain. The similarity between the two linear fit is striking, and we proposed to adopt the same scaling obtained in our previous work also for the grain, without the need to compute the Hessian matrix. This approach will also be valid for other molecules to be considered in future work.

## Conclusion

In the present work, we proposed a new method for building up amorphous water icy cluster models apt to mimic interstellar icy grains. We adopted the semiempirical program GFN-xTB using the GFN2 semiempirical tight-binding Hamiltonian^33^ and the newly developed GFN-FF^34^ force field. A fully automated procedure based on in-house developed python ACO-FROST script is proposed, capable to build up an icy grain by a step by step addition of randomly oriented water molecules. We considered grains of 200 up to 1000 water molecules (with intermediary steps every 100 water molecules). A sequence of geometry relaxations followed by short molecular dynamics steps at 10 K were implemented to resemble the physicochemical of grain formation in the interstellar regions. Careful checks on the icy grain properties (O···H bond length, dangling hydrogen (dH) and oxygen (dO), gyration tensor quantities (gyration radius, asphericity, and relative shape anisotropy), and surface electrostatic potential maps) were carried out to assess the physicochemical features of the model. We showed that the icy grain rapidly reaches a spherical shape, as shown by the gyration tensor, with the dO/dH value showing almost twice as dO than dH sites. This is an important result, implying that the grain is more apt to selectively adsorb hydrogen donor molecules with respect to hydrogen acceptors. Future works will be focused on studying how the dO/dH evolves when heating the grain at higher temperatures (from 10 to 100 K), as experimental works suggested an increasing dO/dH at higher temperature; i.e., the grain becomes deprived of dH sites. We showed that the average hydrogen bond features rapidly reach a common value, as for example, the average number of hydrogen bonds and the above-described dO/dH ratio. As a possible application, we computed the binding energies of NH_3_ at the surface sites of a 200 water grain. By extending the automated procedure adopted for building up the icy grain we set up about 160 different starting sites for the NH_3_ adsorption. The procedure is capable of purging duplicated and ill-defined situations providing a physically sound set of adsorption structures. This approach allows to compute a distribution of binding energies, with a much better statistical significance than previous work by our group^21^ and from other models proposed in the literature. The agreement between our data, even at the relatively cheap GFN2 level of theory, is encouraging when compared with the much expensive *ab initio* data from Ferrero et al.^21^

We believe that the present water cluster can be adopted as a general model to study not only the BE of molecules of interest but diffusion as well, at least with the purpose of rapidly characterizing the diffusion barriers to be refined by more accurate QM:MM calculation. In a forthcoming work, we have adopted and improved the present methodology to obtain a very accurate estimation of the NH_3_ BE distribution with a QM:MM methodology capable to give results at the coupled cluster quality level. The subject and the results will be the content of a future work by our group.

## Supporting Information Available

JSmol rendering of the building up process of a large icy grain and a file containing the GFN2 steps of the 1000 grain building process, the 40 grain structures used for the random seed study, and the 162 final structures of the NH_3_ binding energies computation (a text file with the BE, BE(0), ΔZPE values, and if the binding energy values were included in the final BE(0) distribution of Figure 10 is also included) (ZIP)
Additional details on computational methods and results, including rendered images of obtained structures (PDF)

## Notes

**Data and Software Availability.** The “ACO-FROST” set of scripts is open source and freely available on two github repositories named “ACO-FROST_grain_generator” (https://github.com/aurelegermain/ACO-FROST_grain_generator/) for the grain building process, and ”ACO-FROST_BE-grain” (https://github.com/aurelegermain/ACO-FROST_BE-grain/) for the binding energies computation. The GFN-xTB sets of computational methods are available on the github repository “xtb” (https://github.com/grimme-lab/xtb). All data described in the paper (structure coordinates and alike) are described and made available in the Supporting Information (see specific section for details).
