# Supplementary material for: Computer Generated Realistic Interstellar Icy Grain Models: Physicochemical Properties and Interaction with NH3
Source: ACS Earth Space Chem. 2022 Apr 19;6(5):1286–98. doi: 10.1021/acsearthspacechem.2c00004 (PMC9125691; doi:10.1021/acsearthspacechem.2c00004)
Supplement: Supplementary file 2 — sp2c00004_si_002.pdf [file sp2c00004_si_002.pdf]

Computer Generated Realistic Interstellar Icy Grain Models: Physico-chemical Properties and Interaction with NH_3

Aurèle Germain,[†] Lorenzo Tinacci,[†,‡] Stefano Pantaleone,[¶] Cecilia Ceccarelli,[‡] and Piero Ugliengo[*,†,§]

†*Dipartimento di Chimica, Università degli Studi di Torino, via P. Giuria 7, 10125, Torino, Italy*

‡*Université Grenoble Alpes, CNRS, Institut de Planétologie et d'Astrophysique de Grenoble (IPAG), rue de la Piscine 414, 38000 Grenoble, France*

¶*Dipartimento di Chimica, Biologia e Biotecnologie, Università degli Studi di Perugia, Via Elce di Sotto, 8, 06123, Perugia, Italy*

§*Nanostructured Interfaces and Surfaces (NIS) Centre, Università degli Studi di Torino, via P. Giuria 7, 10125, Torino, Italy*

E-mail: piero.ugliengo@unito.it

Computational details SI

Grain building process SI

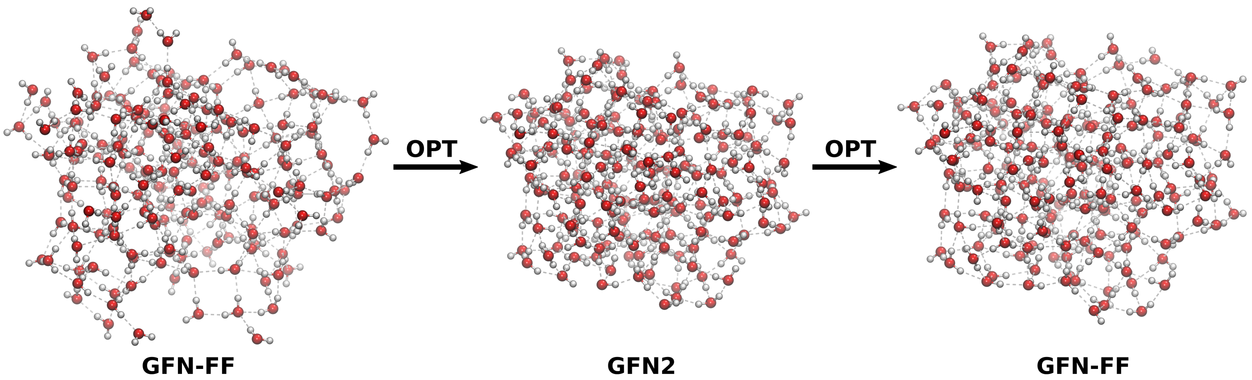

Figure S1: Successive GFN-FF and GFN2 geometry optimisation of an icy grain cluster. The GFN2 optimisation closes the first GFN-FF structure, the GFN-FF optimisation of the GFN2 structure keeps the denser shape but the grain expands due to the higher hydrogen bond length of GFN-FF. Each image have the same scale.

https://aurelegermain.github.io/JSmol_grain/movie_grain_1000.html

Figure S2: Link to a video of a 1000 water molecules grain building process (The video can take several minutes to load).

Binding energy sampling SI

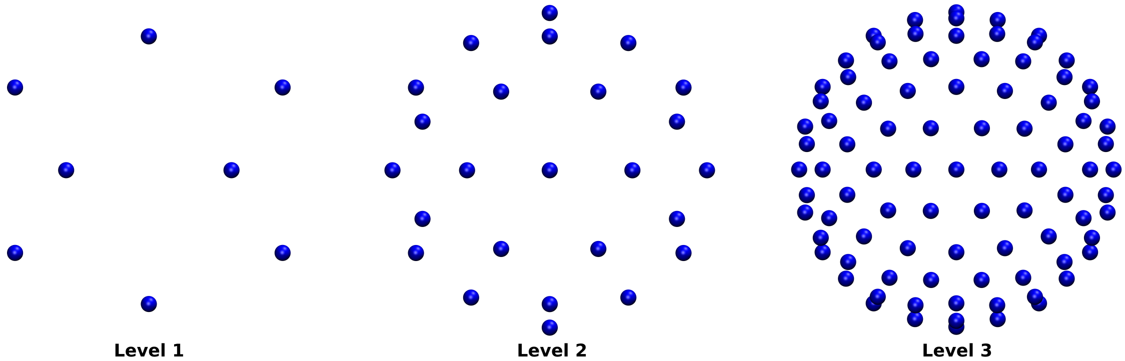

Figure S3: 3 different level of grids. Level 1: 12 vertex. Level 2: 42 vertex. Level 3: 162 vertex.

Results SI

Icy grain model SI

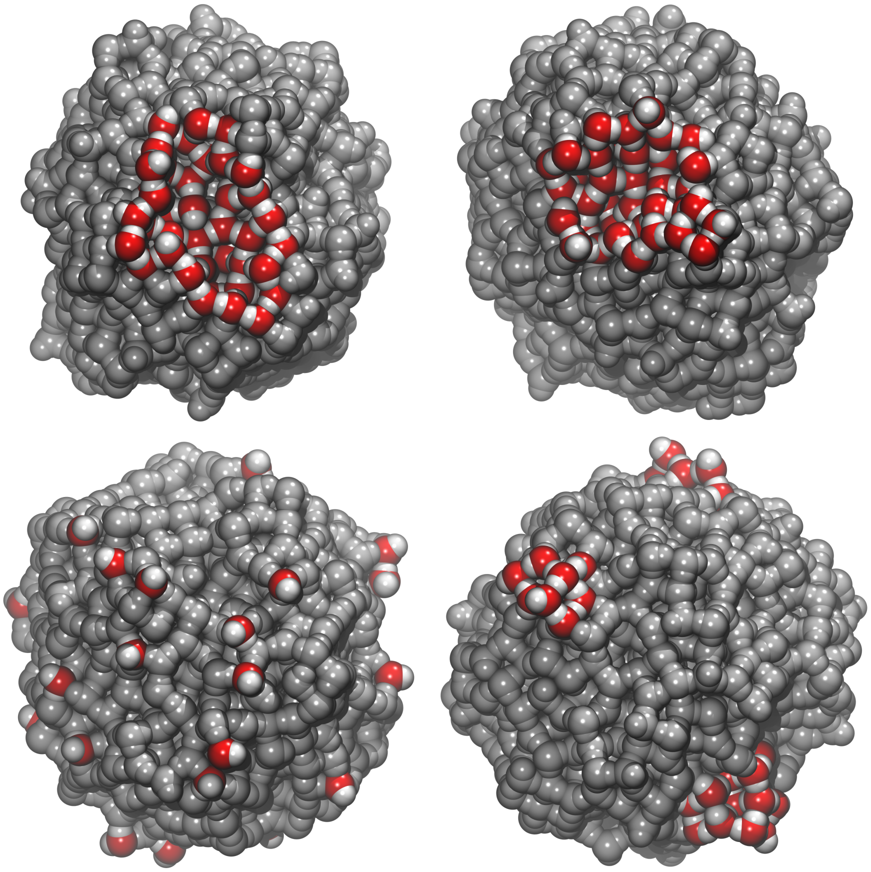

Figure S4: Top: Two cavities. Bottom: Dangling hydrogens on the grain surface on the left, and several protruding spots on the right.

Binding energy sampling SI

From the fixed grain to the unfixing of the water molecules in the 5Å radius we observe an increase of almost 10 kJ/mol in the average BE values. This is due in part to the relaxation of the water molecules present in the 5Å radius around NH_3 after being unfixed, as can be seen in Fig. S6 where NH_3 is able to make an hydrogen bond with a dangling oxygen of the grain after relaxation. In the few cases where NH_3 works as an hydrogen bond donor, the

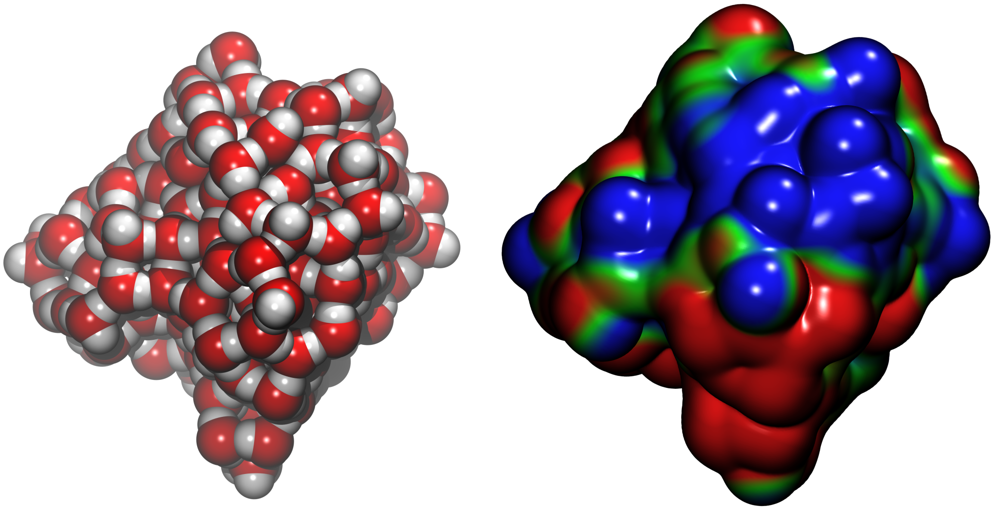

Figure S5: Left: Image of the 200 water molecule icy grain Right: Electostatic potential surface map of the same grain. In blue H-bond donor (dangling Hydrogen) and red H-bond acceptor (dangling Oxygen).

weak interaction and the ensuing high distance between the molecule and the grain surface (as visible on Fig. 10 of the main text) results in a quasi non-existent change in the grain structure after unfixing the 5Å radius and thus a very minute change in binding energies values for the smaller BE. As a consequence we obtain a more spread out BE distribution for the unfixed binding energies than for the fixed ones.

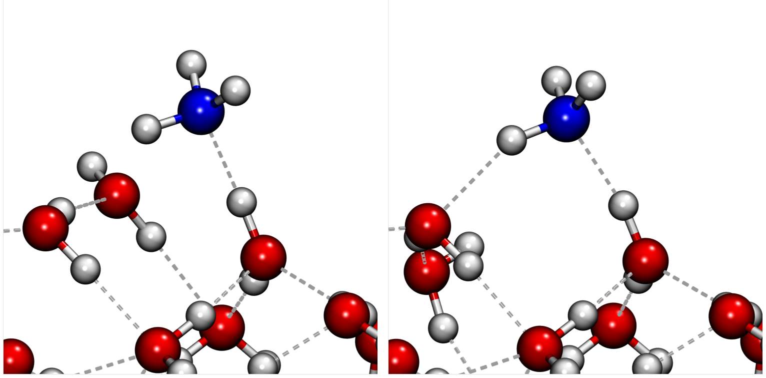

Figure S6: Left: First geometry optimisation with the grain structure entirely fixed. Right: Second geometry optimisation, after relaxation of the water molecules contained inside the 5 Å radius around NH_3.
